# Chromosome End Repair and Genome Stability in *Plasmodium falciparum*

**DOI:** 10.1128/mBio.00547-17

**Published:** 2017-08-08

**Authors:** Susannah F. Calhoun, Jake Reed, Noah Alexander, Christopher E. Mason, Kirk W. Deitsch, Laura A. Kirkman

**Affiliations:** a Department of Microbiology and Immunology, Weill Medical College of Cornell University, New York, New York, USA; b Department of Physiology and Biophysics, Weill Medical College of Cornell University, New York, New York, USA; c Department of Medicine, Division of Infectious Diseases, Weill Medical College of Cornell University, New York, New York, USA; University of Pittsburgh

**Keywords:** *Plasmodium falciparum*, chromosome stability, *de novo* telomere addition, gene conversion, homologous recombination, telomere healing

## Abstract

The human malaria parasite *Plasmodium falciparum* replicates within circulating red blood cells, where it is subjected to conditions that frequently cause DNA damage. The repair of DNA double-stranded breaks (DSBs) is thought to rely almost exclusively on homologous recombination (HR), due to a lack of efficient nonhomologous end joining. However, given that the parasite is haploid during this stage of its life cycle, the mechanisms involved in maintaining genome stability are poorly understood. Of particular interest are the subtelomeric regions of the chromosomes, which contain the majority of the multicopy variant antigen-encoding genes responsible for virulence and disease severity. Here, we show that parasites utilize a competitive balance between *de novo* telomere addition, also called “telomere healing,” and HR to stabilize chromosome ends. Products of both repair pathways were observed in response to DSBs that occurred spontaneously during routine *in vitro* culture or resulted from experimentally induced DSBs, demonstrating that both pathways are active in repairing DSBs within subtelomeric regions and that the pathway utilized was determined by the DNA sequences immediately surrounding the break. In combination, these two repair pathways enable parasites to efficiently maintain chromosome stability while also contributing to the generation of genetic diversity.

## IMPORTANCE

Malaria is a major global health threat, causing approximately 430,000 deaths annually. This mosquito-transmitted disease is caused by *Plasmodium* parasites, with infection with the species *Plasmodium falciparum* being the most lethal. Mechanisms underlying DNA repair and maintenance of genome integrity in *P. falciparum* are not well understood and represent a gap in our understanding of how parasites survive the hostile environment of their vertebrate and insect hosts. Our work examines DNA repair in real time by using single-molecule real-time (SMRT) sequencing focused on the subtelomeric regions of the genome that harbor the multicopy gene families important for virulence and the maintenance of infection. We show that parasites utilize two competing molecular mechanisms to repair double-strand breaks, homologous recombination and *de novo* telomere addition, with the pathway used being determined by the surrounding DNA sequence. In combination, these two pathways balance the need to maintain genome stability with the selective advantage of generating antigenic diversity.

## INTRODUCTION

Malaria, a mosquito-transmitted infectious disease, continues to pose a major challenge for health care systems in many parts of the developing world (1). The disease is caused by various species of the *Plasmodium* genus of protozoan parasites, members of the *Apicomplexa* family of obligate parasites that also includes *Toxoplasma*, *Babesia*, *Cryptosporidium*, and *Theileria* species. The pathogenic stage of malaria occurs when parasites invade and replicate within the red blood cells (RBCs) of their vertebrate hosts, causing cycles of fever and chills as well as severe anemia. *Plasmodium falciparum*, the species responsible for the most virulent form of the disease, also causes infected RBCs to become cytoadhesive, resulting in sequestration within the postcapillary vasculature, leading to circulatory disruption and many of the severe symptoms of the disease, including cerebral malaria and complications during pregnancy (2). While multiplying within the RBCs, the parasites are haploid, replicating through a process called schizogony in which multiple rounds of nuclear division lead to a multinucleated cell containing 16 to 32 individual merozoites. These are released upon lysis of the host cell, enabling them to invade new RBCs and reinitiate the cycle. Throughout this process, parasites are confronted with numerous conditions that can cause DNA damage, including the products of metabolism and hemoglobin digestion, components of the host immune response, and oxidative damage resulting from exposure to antimalarial drugs (3–5). The success of these parasites therefore depends on their ability to efficiently repair DNA damage and maintain genome integrity.

**FIG 1:**
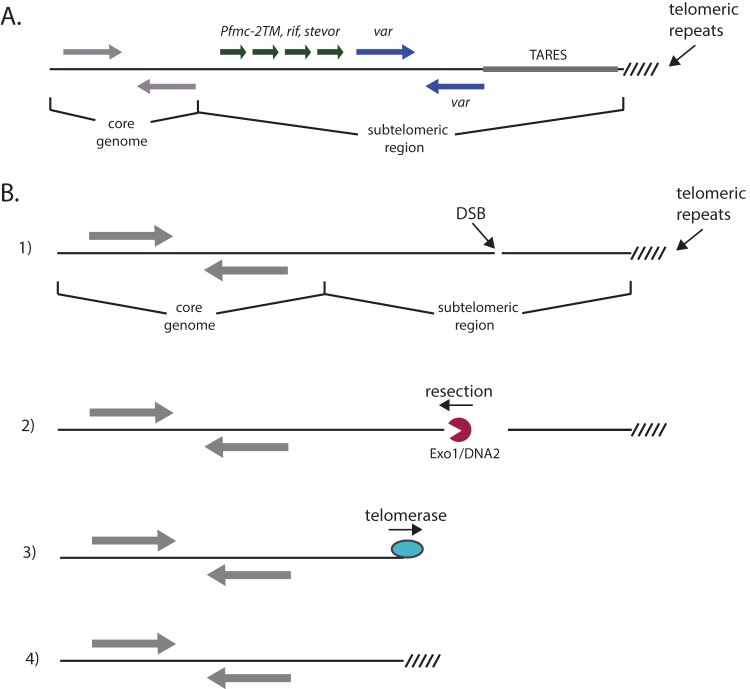
Repair of double-strand breaks within subtelomeric chromosomal regions. (A) The typical structure of the subtelomeric regions of the chromosomes of *P. falciparum*. The core genome contains primarily single-copy housekeeping genes (gray), while the subtelomeric regions consists of large arrays of variant antigen-encoding genes of the *var* (blue), *rif*, *stevor*, and *Pfmc-2TM* (green) families. TAREs are positioned between the variant gene families and the telomere repeats. (B) The steps of *de novo* telomere addition, also called telomere healing. Step 1: typical chromosomes can be divided into a core genome containing housekeeping genes, telomere repeats at the extreme end of the chromosome, and intervening subtelomeric regions. A DSB within the subtelomeric region can be repaired by telomere healing. Step 2: the DSB is recognized by protein complexes that include exonuclease activity. In model organisms, both Exo1 and DNA2 have been implicated in the resection of DNA away from the telomere, revealing a single-stranded 3′ end. Step 3: when a single-strand sequence is revealed that can anneal to the template RNA of the telomerase complex, telomerase activity extends from the break, placing telomere repeats directly at this site. Step 4: repeated rounds of telomere addition result in a stable telomere and maintain genome integrity.

The *P. falciparum* genome is arranged into 14 linear chromosomes constituting a total of approximately 23 Mb (6). The contents of each individual chromosome are organized similarly, with single-copy genes responsible for replication and progression through the cell cycle within the central regions and members of the large, multicopy, clonally variant gene families found in lengthy arrays within the subtelomeric domains. Flanking the variant genes are conserved telomere-associated repeat elements (TAREs), which typically extend for 10 to 30 kb, followed by the actual chromosome end, which consists of typical telomeric heptad repeats maintained by telomerase (Fig. 1A) (7). The subtelomeric domains of *P. falciparum* are of particular interest for understanding parasite biology, since the clonally variant gene families that reside there are the primary virulence determinants and their varied expression results in antigenic variation, the process that enables parasites to perpetuate long-term, chronic infections. These regions contain hundreds of genes from several gene families, such as *var*, *stevor*, *rifin*, *Pfmc-2TM*, *FIKK* and *ACS*, which are maintained in a unique chromatin structure that is marked by the histone modifications H3K9me3 and H3K36me3 (8–10). Within the nucleus, these regions cluster at the nuclear periphery, an arrangement that is thought to facilitate recombination between members of the multicopy gene families that reside on different chromosomes, thereby generating diversity (11, 12). Given the importance of these chromosomal regions for pathogenesis, understanding how they are replicated, maintained, and repaired is of considerable interest.

DNA double-strand breaks (DSBs) are an especially severe form of DNA damage that must be repaired for a cell to remain viable and to replicate. Eukaryotic cells typically rely on two distinct pathways to repair such breaks, homologous recombination (HR) and nonhomologous end joining (NHEJ). While NHEJ can ligate the broken ends of the DNA back together to efficiently repair a break, it often results in small deletions at the site of ligation and can therefore be mutagenic (13). HR is a more accurate method of repair since it copies information from homologous sequences elsewhere in the genome, typically the other allele in a diploid cell, thereby avoiding any insertion or deletion at the site of the break. HR, however, requires a homologous region of high sequence identity (typically greater than 98%) to serve as the template for repair (14). The asexual stages of *P. falciparum* represent a unique organism for the study of DSB repair, since they are haploid and thus generally lack homologous sequences to serve as the templates for HR, and yet they are missing the canonical NHEJ pathway. An alternative NHEJ pathway based on microhomology has been described, although it appears to be quite inefficient (15, 16). Therefore, how malaria parasites respond to DSBs and efficiently maintain genome integrity is unclear. Characterization of important enzymes involved in HR demonstrated that this pathway is functionally conserved (17–19). The subtelomeric regions, given their size, extensive gene content, semirepetitive nature, and unique chromatin structure, represent a particularly interesting region of the genome for studying DSB repair.

In addition to HR and NHEJ, eukaryotic organisms can also stabilize DSBs occurring at the chromosome ends through the action of telomerase, the enzyme that maintains the telomeric repeats at this specialized chromosomal location. This repair pathway, called *de novo* telomere addition, or “telomere healing,” involves the recruitment of the telomerase complex directly to the site of the break (20, 21). The DNA strand is resected until a region of high TG content is encountered, which is thought to “seed” telomerase and enable it to incorporate telomeric repeats at the site of a DNA break and resection (Fig. 1B). This results in the creation of a functional telomere, thereby stabilizing the chromosome and maintaining genome integrity, albeit with a deletion of the region of the chromosome between the DSB and the original telomere and loss of the intervening genetic information. Telomere healing is thought to compete with HR and NHEJ for repair of DSBs, with the pathway of repair depending on the chromosomal environment in which the break occurs (20). Telomere healing has been described for a number of eukaryotic organisms, and the addition of telomeric repeats to the ends of broken chromosomes has been described for *P. falciparum* (22–24), indicating that this mechanism of repair is conserved. However, the details of telomere healing in malaria parasites and how this pathway integrates with HR and alternative NHEJ have not been studied extensively.

Given the general chromosome structure of *P. falciparum* (Fig. 1A), telomere healing could repair DSBs that occur anywhere within the extensive subtelomeric regions between the chromosome ends and the internal, highly conserved regions of the genome. Genes within these subtelomeric regions are necessary for host-pathogen interactions but are not required for viability in cultured parasites; thus, deletions of subtelomeric regions are tolerated and can be efficiently recovered and analyzed. The semirepetitive nature of these regions suggests that DSBs could also potentially be repaired by HR, allowing the study of both pathways as they repair breaks within this chromosomal environment. Detailed sequence examination of subtelomeric domains has been difficult in the past due to the repetitive nature of the sequences, making assemblies of these regions from short sequence reads problematic. However, newer technologies, including those that utilize single-molecule real-time (SMRT) sequencing, enable the confident assembly of complete subtelomeric regions, allowing us to examine in detail DSB repair in these regions of the genome. We applied this technology to study the repair of spontaneous DSBs that occur within subtelomeric regions during *in vitro* culture as well as breaks induced randomly through exposure to ionizing radiation.

Our analysis indicated that both telomere healing and gene conversion through HR can repair DSBs within subtelomeric domains. Telomere healing was the most common type of repair we observed, and which pathway was utilized was strictly determined by the sequence surrounding the break point. Considering the repetitive nature of these chromosomal regions, subtelomeric deletions resulting from telomere healing events could be followed by subsequent gene conversion through HR, thus reestablishing the typical subtelomeric structure and maintaining the overall parasite’s chromosome organization. We conclude that both repair pathways therefore work in tandem to maintain genome integrity and preserve the complement of clonally variant genes found within the genomes of *P. falciparum* isolates.

## RESULTS

### Extensive telomere healing is observed in cultured parasites.

While telomere healing has been described for *P. falciparum* (22–24), how commonly it occurs within the 28 subtelomeric domains of the parasite’s genome has not been closely examined. Telomere healing has been most extensively studied in *Saccharomyces cerevisiae*, in which telomerase displays a strong preference for TG-rich sequences to initiate synthesis of repeats (25, 26). If *P. falciparum* telomerase has a similar preference for TG-rich sequences to initiate synthesis of repeats, then one is likely to observe telomeric repeats fused to the coding regions of truncated genes at the new chromosome ends, as coding regions are significantly enriched in Cs and Gs compared to noncoding regions of the genome.

**FIG 2:**
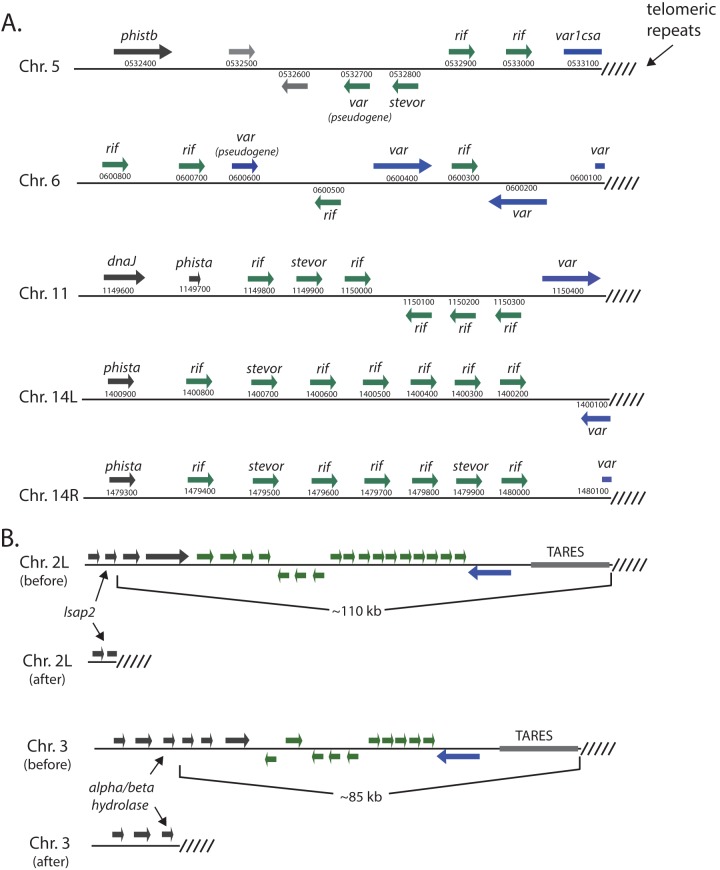
Telomere healing events identified in the genome of the parasite 3D7. (A) The five telomere healing events identified in the genome of the reference 3D7 sequence. The chromosome number is shown along with the gene structure of each subtelomeric region. For each gene, the gene family is indicated as well as the annotation number (provided by PlasmoDB.org). (B) Two additional telomere healing events identified in a clonal line of 3D7. The event that occurred on chromosome 2 (top) resulted in deletion of ~110 kb and led to truncation of the *lsap2* gene (PF3D7_0202100), while the healing event on chromosome 3 resulted in loss of ~85 kb and added telomere repeats just downstream of a gene encoding a predicted alpha/beta-hydrolase (PF3D7_0301300).

As a first step to determine if telomere healing is common in cultured *P. falciparum* and to obtain a baseline for our experimental analysis, we analyzed the sequence of all 28 chromosome ends provided in the reference genome sequence of 3D7 (Plasmodb.org). Five chromosome ends display the hallmarks of telomere healing, including the absence of TAREs and telomeric repeat sequences fused directly into or next to protein coding regions. In four of these, on chromosomes 5 and 6 and both ends of chromosome 14, the telomeric repeats are found within the coding region of a truncated *var* gene, creating a pseudogene. In the fifth example on chromosome 11, the telomeric repeats are found immediately downstream of an intact *var* gene (Fig. 2A).

Next, we investigated if telomere healing events occurred in parasites cultivated in the laboratory; we generated several subclones from a stock of 3D7 parasites that had been grown in culture for several years in our laboratory and chose one for whole-genome sequencing. To determine the sequence of the chromosome ends and to avoid difficulties in assembling the repetitive structure of subtelomeric domains from the short sequence reads typically derived from most methods of high-throughput sequencing, we instead utilized SMRT sequencing, which can yield single contiguous reads of 10 to 20 kb (27, 28). Using this method, we verified the five telomere healing events recognized in the reference genome sequence and also identified two additional novel DNA sequences consistent with telomere healing (Fig. 2B; see also Fig. S1, S3, and S4 in the supplemental material). The first, on chromosome 2, resulted in the deletion of approximately 110 kb, including the entire subtelomeric domain and leading to the insertion of telomeric repeats within the *lsap2* gene (PF3D7_0202100). The second occurred on chromosome 3 and similarly deleted all of the subtelomeric domain (~85 kb), inserting telomeric repeats just downstream of a gene encoding a putative alpha/beta-hydrolase (PF3D7_0301300). These two events provided us with a “before and after” picture of telomere healing and further indicated that this is a common mechanism of DSB repair within the subtelomeric regions of *P. falciparum*.

10.1128/mBio.00547-17.1 FIG S1  Sequence read “pile-ups” displaying the detection of telomere healing events on chromosomes 2 and 3. These events are displayed schematically in Fig. 2B of the main text. For both chromosomes, the reads obtained from the irradiated clone are shown on the top, and the reads obtained for the nonirradiated clone are shown on the bottom. All reads were aligned with the 3D7 reference sequence obtained from Plasmodb.org. The sites of telomere healing are denoted with a red arrow. The subtelomeric deletions were detectable in both the irradiated and nonirradiated clones, indicating that these two healing events occurred during culture of the parent line prior to exposure to X-ray irradiation. Reads that aligned within the deleted regions in the irradiated line coincide with TAREs that are shared between many subtelomeric domains. Download FIG S1, PDF file, 0.5 MB. Copyright © 2017 Calhoun et al. 2017 Calhoun et al. This content is distributed under the terms of the Creative Commons Attribution 4.0 International license.

**FIG 3:**
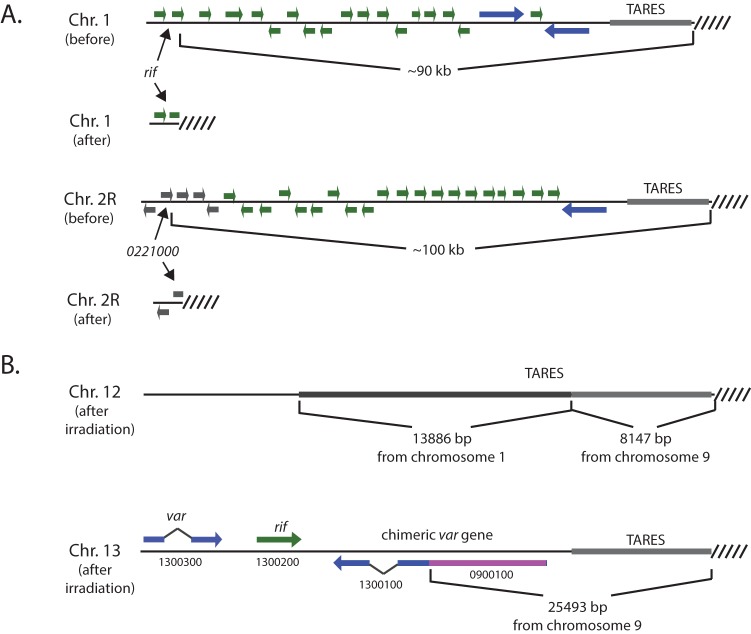
Double-strand break repair within subtelomeric regions in response to X-ray irradiation. (A) Two products of telomere healing are shown. On chromosome 1 (top), telomere repeats were inserted into the coding region of the *rif* gene (Pf3D7_0101900) and on chromosome 2 (bottom), in which telomere repeats were inserted into the hypothetical gene Pf3D7_0221000. These events resulted in subtelomeric deletion of ~90 and ~100 kb, respectively. (B) Three products of repair by homologous recombination were also identified, two on chromosome 12 (top) and one on chromosome 13 (bottom). The two events on chromosome 12 resulted in the insertion of subtelomeric sequences from chromosomes 1 and 9; however, no coding regions were altered. The event on chromosome 13 resulted in the insertion of 25,493 bp of sequence from chromosome 9 and led to the creation of a new *var* gene that is a chimera of Pf3D7_1300100 (blue) and Pf3D7_0900100 (pink).

10.1128/mBio.00547-17.2 FIG S2  Sequence read “pile-ups,” displaying the detection of telomere healing events on chromosomes 1 and 2. These events are displayed schematically in Fig. 3A of the main text. For both chromosomes, the reads obtained from the irradiated clone are shown on the top and the reads obtained from the nonirradiated clone are shown on the bottom. All reads were aligned with the 3D7 reference sequence obtained from Plasmodb.org. The sites of telomere healing in the irradiated clone are denoted with a red arrow. Reads that aligned within the deleted regions in the irradiated line coincide with TAREs that are shared between many subtelomeric domains. Download FIG S2, PDF file, 0.7 MB. Copyright © 2017 Calhoun et al. 2017 Calhoun et al. This content is distributed under the terms of the Creative Commons Attribution 4.0 International license.

10.1128/mBio.00547-17.3 FIG S3  Assembled sequence showing the telomere healing event associated with the end of chromosome 2L, as shown schematically in Fig. 2B of the main text. The coding region of the gene *lsap2* is shown in black text, while the telomeric repeats are shown in blue. Download FIG S3, PDF file, 0.4 MB. Copyright © 2017 Calhoun et al. 2017 Calhoun et al. This content is distributed under the terms of the Creative Commons Attribution 4.0 International license.

10.1128/mBio.00547-17.4 FIG S4  Assembled sequence showing the telomere healing event associated with the end of chromosome 3, as shown schematically in Fig. 2B of the main text. The coding region of the alpha/beta-hydrolase gene is shown in black text, while the telomeric repeats are shown in blue. A short stretch of subtelomeric DNA between the coding region and the telomere repeats is shown in purple text. Download FIG S4, PDF file, 0.4 MB. Copyright © 2017 Calhoun et al. 2017 Calhoun et al. This content is distributed under the terms of the Creative Commons Attribution 4.0 International license.

### Inducement of DSBs and repair in cultured parasites by X-ray irradiation.

To more directly observe DSB repair within subtelomeric domains, we chose to induce random DSBs by exposing parasite cultures to X-ray. X-rays are known to cause DSBs without sequence bias, and such breaks must be repaired for parasite viability. Thus, by selecting for viable parasites after near-lethal exposure to X-rays, we hoped to detect and analyze additional examples of DSBs within subtelomeric regions. The subclone of 3D7 previously used for SMRT sequencing was exposed to increasing amounts of X-ray radiation to determine the level of exposure that would lead to significant widespread DNA damage yet allow for parasites to repair, recover, and propagate. Exposure to 100 Gy resulted in significant lethality; however, viable parasites grew from irradiated cultures within 10 to 12 days after exposure. Parasites were exposed to 100 Gy three times consecutively, allowing the parasites to recover normal growth after each irradiation. Subclones were then isolated by limiting dilution, and one clone was subjected to whole-genome sequencing using the SMRT methodology. Analysis of the genome sequence identified two additional examples of telomere healing, one on chromosome 1 that led to a deletion of ~90 kb and resulted in the insertion of telomeric repeats into a *rifin* gene (Pf3D7_0101900) and a second on chromosome 2 that led to deletion of ~100 kb and resulted in the insertion of telomeric repeats just downstream of the hypothetical gene Pf3D7_0221000 (Fig. 3A; Fig. S2, S5, and S6). This clone of 3D7 now carries significant truncations within 9 of its 28 subtelomeric regions.

10.1128/mBio.00547-17.5 FIG S5  Assembled sequence showing the telomere healing event associated with the end of chromosome 1, as shown schematically in Fig. 3A of the main text. The coding region of a *rif* gene is shown in black text, while the telomeric repeats are shown in blue. Download FIG S5, PDF file, 0.4 MB. Copyright © 2017 Calhoun et al. 2017 Calhoun et al. This content is distributed under the terms of the Creative Commons Attribution 4.0 International license.

10.1128/mBio.00547-17.6 FIG S6  Assembled sequence showing the telomere healing event associated with the end of chromosome 2R, as shown schematically in Fig. 3A of the main text. The coding region of Pf3D7_0221000 is shown in black text, while the telomeric repeats are shown in blue. Download FIG S6, PDF file, 0.4 MB. Copyright © 2017 Calhoun et al. 2017 Calhoun et al. This content is distributed under the terms of the Creative Commons Attribution 4.0 International license.

In addition to the two new examples of telomere healing identified for this clone, we also detected three examples of recombination events that likely resulted from HR. One of the subtelomeric regions of chromosome 13 is a hybrid sequence in which the original subtelomeric region has been deleted and replaced by ~25,500 bp, including the telomeric repeats, from one of the subtelomeric regions of chromosome 9. The breakpoint of the recombination event occurred within the coding region of a *var* gene, creating a new *var* gene that is a chimera of PF3D7_0900100 and PF3D7_1300100 (Fig. 3B). A more complex product of recombination was identified near one end of chromosome 12. The first ~8,100 bp, including the telomeric repeats, was derived from one of the subtelomeric domains of chromosome 9 and, given that both ends of chromosome 9 remain unchanged, this appears to be the result of a gene conversion event. This fragment is fused to 13,886 bp of sequence identical to a region within one of the subtelomeric domains of chromosome 1. This sequence is within the portion of chromosome 1 that is now deleted in this clone, indicating that it was transposed into the subtelomeric region of chromosome 12 prior to its deletion from chromosome 1 (Fig. 3B). This could have resulted from either gene conversion or reciprocal recombination. These data indicate that DSBs that occur within subtelomeric domains can be repaired either by HR or telomeric healing and that the repair process can generate new *var* genes.

### Sequence preference for insertion of telomeric repeats by telomerase.

**FIG 4:**
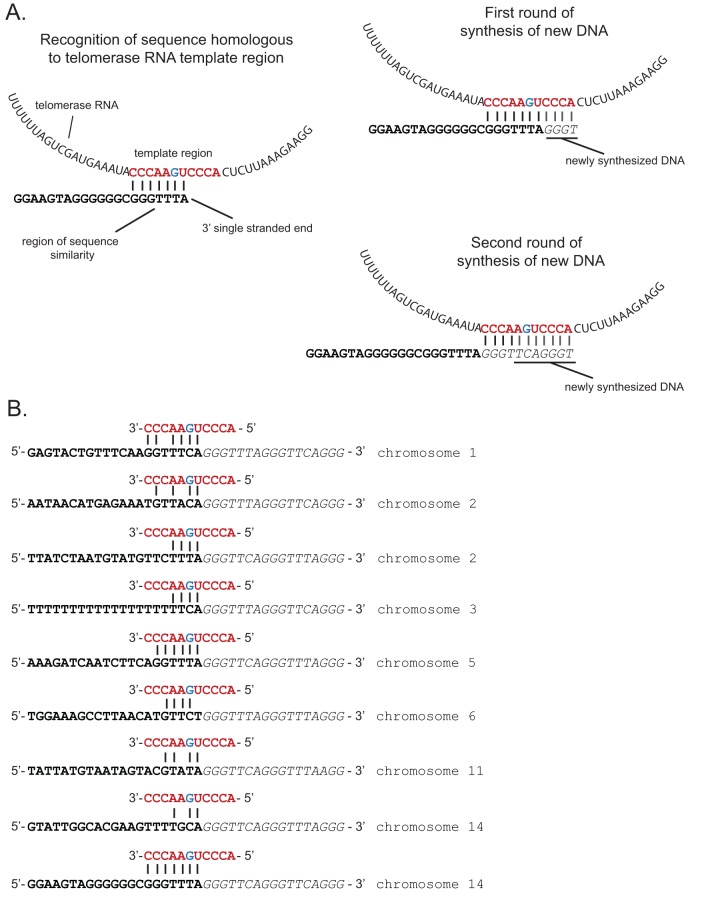
Sequence preference for telomere healing. (A) Model for telomere repeat additions at the site of a DSB. After exonuclease resection, a 3′ single-strand overhang is revealed. Single-stranded regions of sequence similarity can anneal to the template region of telomerase RNA (left). Telomerase activity can then extend by adding telomere repeat sequences directly to the chromosome end (right). Multiple rounds of repeat addition result in a lengthy telomere repeat region. (B) The nine examples of telomere healing identified in this study. Bold sequence portions indicate the original region of the chromosome, while italicized letters indicate the repeat sequences added by telomerase. The red sequence shows the template region of telomerase RNA and hypothetical annealing to the sequence where the healing event occurred. The blue G within the template indicates the base pair that is thought to specify either a C or T.

A model for telomere healing in higher eukaryotic cells has been derived from extensive experiments conducted primarily in yeast. When a DSB forms within a subtelomeric region, the DNA is initially resected by one of two exonucleolytic pathways (Exo1 or Dna2/Sgs1), revealing a region of 3′ single-stranded DNA (29). The resection continues until a sequence is encountered that can anneal to the template region of telomerase RNA. This allows telomerase to begin synthesizing the telomeric repeats directly at the end of the chromosome, continuing for multiple rounds of DNA synthesis and resulting in a functional telomere that can stabilize the chromosome end (Fig. 4A). As predicted by this model, telomere healing is initiated at sites that display sequences complementary to the template region of telomerase RNA. In yeast, these sites almost always include GT, TG, or CG dinucleotides at the site where telomerase initiates synthesis of telomere repeats (25, 26). In addition, the flanking region also plays a role in determining where the new telomere is synthesized, presumably by influencing how efficiently telomerase is recruited to the chromosome end (30). In *S. cerevisiae*, stretches of TG repeats between 22 and 250 bp effectively recruit telomerase for healing, but stretches either longer or shorter are repaired much less efficiently (31–34). Proximal enhancer sequences that can function as binding sites for proteins that associate with the telomerase complex can also increase the efficiency of telomere healing (30). Sites of telomere healing in *P. falciparum* were previously identified within genes that are known to be commonly disrupted in cultured parasites, presumably because such deletion events provide a growth advantage *in vitro* (22, 24); the ability of *P. falciparum* telomerase obtained from nuclear extracts to extend from specific sequences *in vitro* was also investigated (23). The identification of 9 independent telomere healing events in our sequence data, including events that we induced with radiation exposure, allowed us to investigate in more detail the properties at the site of a DSB that contribute to telomere healing in *P. falciparum* and whether they differ from sites that are instead repaired by HR.

The sequence of the telomerase RNA template region for several *Plasmodium* species was predicted bioinformatically by Chakrabarti et al. (35). This template sequence is known to be species specific and, unlike the equivalent sequence in yeast, the *P. falciparum* sequence does not consist exclusively of As and Cs and instead includes two additional bases (UG) within the sequence (5′-ACCCUGAACCC-3′). Interestingly, given that the two major telomeric repeat sequences are 5′-TTCAGGG-3′ and 5′-TTTAGGG-3′, the G within the template sequence appears to specify either C or T. The sites where telomeric repeats were added to the chromosome ends for all nine telomere healing events identified in our sequence datasets are shown in Fig. 4B. As can be easily discerned, the sites where repair was initiated in all nine sequences displayed the ability to anneal to the telomerase template region at precisely the same position; thus, in all cases the first bases added to the new telomere were GGGTT. These data indicate that telomere healing in *P. falciparum* likely involves the same mechanism described for yeast, yet the sequence of the telomerase RNA template explains why the site of telomere repeat addition diverges from the GT, TG, or CG preference described for *S. cerevisiae*. Examination of the sequences upstream of the newly added telomere repeats did not identify any discernible motifs or compositional bias, indicating either that proximal enhancers like those identified in yeast do not exist in *P. falciparum* or that recruitment of the telomerase complex is not influenced by the primary sequence.

### Choice of repair pathway: HR versus telomere healing.

In most extensively studied organisms, DSB repair in most regions of the genome results from competition between the two primary repair pathways, HR and NHEJ. Within subtelomeric regions of the genome, telomere healing can serve as a third potential repair pathway. The pathway that is ultimately chosen depends on multiple factors and is often species specific (20, 21). In yeast, the likelihood of telomere healing occurring at a specific break appears to depend on how efficiently the telomerase complex is recruited to the site of the break, which in turn depends on the DNA sequence immediately upstream of where the repair event occurs (20). Telomerase can then initiate the addition of telomeric repeats when 3 to 6 bp of DNA anneals to the telomerase RNA template (Fig. 4) (25). Telomerase has been shown to be recruited by either Cdc13 or the Ku70/80 complex (26, 36–38), and a proximal Cdc13 binding sequence was shown to greatly increase the likelihood of a healing event at a particular chromosomal position (30). Malaria parasites lack the above-mentioned DNA repair proteins; thus, what determines whether HR or telomere healing occurs at the site of a subtelomeric break is not known.

**FIG 5:**
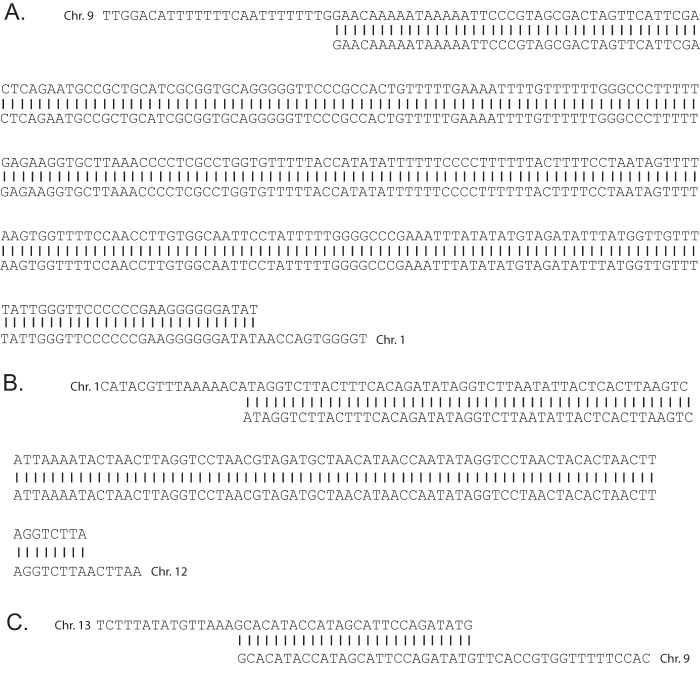
The sequences surrounding the break points of the three examples of DSB repair by homologous recombination identified in this study. (A and B) The recombination events that occurred within one of the subtelomeric regions of chromosome 12. The first (A) occurred within a region of 296 bp of sequence identity between chromosomes 9 and 1, while the second (B) occurred within a region of 127 bp of sequence identity between chromosomes 1 and 12. (C) A region of 27 bp of sequence identity spanning the break point of the recombination event found within a subtelomeric region of chromosome 13, leading to the creation of a chimeric *var* gene.

Previous work has shown that efficient HR within a nonsubtelomeric region of the genome requires near-complete sequence identity between the sequence surrounding the break and the template used for repair (15). If this property also applies to HR within subtelomeric regions, the use of HR might be limited only to breaks that occur within stretches of sequence that are duplicated with near-complete sequence identity elsewhere in the genome. To investigate this possibility, we identified the sequences immediately surrounding the break points of the three products of HR that we obtained from our irradiated clone and performed BLAST searches to determine if these regions are duplicated elsewhere in the genome. Indeed, in all three cases there were regions of perfect sequence identity surrounding the site of the recombination event. These stretches of 100% identity extended for 296, 127, and 27 bp within the sequences of the recombining chromosomes (Fig. 5). In contrast, BLAST searches using stretches of sequence at the nine sites of telomere healing indicated that these regions are unique within the genome, with no identifiable stretches of sequence identity at the site of telomere addition. These data are consistent with a model in which HR is the dominant pathway of DSB repair throughout the genome, including within the subtelomeric regions. However, the strict requirement for extensive sequence identity prevents this pathway from repairing breaks that occur within unique sequences, thus allowing telomere healing to occur when sequences similar to the telomerase template RNA are exposed during resection of the DNA strand.

## DISCUSSION

Telomere healing appears to be a conserved mechanism for stabilizing chromosomes in the event of a DSB that occurs within a subtelomeric domain. The basic machinery involved in telomere healing is likely shared between malaria parasites and model eukaryotes. In yeast, either the Exo1 or DNA2/Sgs1 pathways mediate DNA resection from the site of the break. Orthologues to both of these enzymes appear to be encoded within the *P. falciparum* genome (PF3D7_0725000 and PF3D7_1010200, respectively). In addition, telomerase reverse transcriptase (PfTERT) has also been identified and displays the expected motifs, though with many insertions of stretches of basic amino acid sequences, as is often seen in *Plasmodium* proteins (39). However, our work revealed some unique aspects to telomere healing in *P. falciparum*. The strong preference for stretches of TG repeats at the site of telomere addition that is observed in yeast was not seen in *P. falciparum*, nor was any proximal enhancer sequence detected. The different sequence preference likely results from differences in the template region of telomerase RNA, which in yeast consists exclusively of AC base pairs, while in *P. falciparum* this sequence also includes a GU dinucleotide. The apparent lack of any preference in the sequence immediately flanking the site of repair might indicate more significant evolutionary divergence in how telomerase is recruited. Organism-specific proteins at telomeres have been characterized in model organisms and in African trypanosomes, and they indicate there can be significant evolutionary divergence (40). Proteins known to play important roles in telomerase function and telomere stabilization in other organisms, such as TRF, Cdc13, Rad 52, and POT1, could not be identified in the *P. falciparum* genome using standard bioinformatics approaches, and thus the recruitment, retention, and function of telomerase in *P. falciparum* is likely to have unique elements, such as the recently characterized protein PfTRZ. This protein was found to be a functional homologue to the transcription factor TFIIIA, yet it is associated with parasite telomeres and has a role in telomere maintenance (41).

The subtelomeric domains of the chromosomes of *P. falciparum* are of significant research interest due to the large multicopy gene families that reside within these regions. The unique structure of the chromatin found here has been shown to play a role in regulating clonally variant expression, thereby facilitating the process of antigenic variation and immune system avoidance (42). The positions of these large, semiredundant gene families within the subtelomeric domains of most or all of the chromosomes mean that these genomic regions share significant blocks of sequence identity. In addition, the clustering of these regions at the nuclear periphery makes them prime substrates for HR in the event of a DSB (11). This unique genomic organization, combined with the absence of canonical NHEJ, provides a simple mechanism that drives the generation of chimeric genes and thus the vast diversity of these gene families that is observed in the field. Indeed, a chimeric *var* gene was readily generated over the course of the experiments described here.

**FIG 6:**
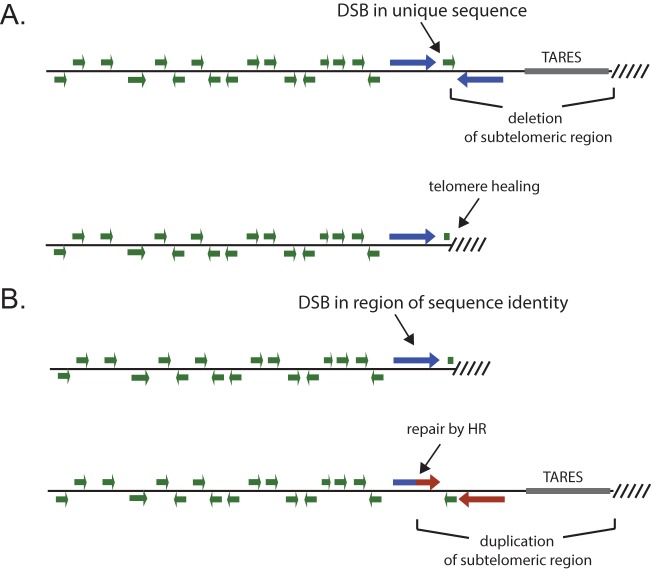
Model for the contribution of both telomere healing and homologous recombination in maintaining chromosome end stability in *P. falciparum*. (A) The occurrence of a DSB at a site of unique sequence within a subtelomeric region is stabilized by telomere healing. This results in a substantial deletion of the subtelomeric domain, including members of multicopy gene families and TAREs. (B) A subsequent DSB within a region that shares sequence identity with subtelomeric regions from other chromosomes can be repaired by HR, leading to reestablishment of the normal subtelomeric structure, including a full complement of multicopy genes and TAREs. Repair by HR can also result in chimeric genes, thereby contributing to the generation of diversity within the multicopy gene families.

While repair of DSBs within subtelomeric domains by HR is the likely source of diversity within the multicopy gene families, HR appears to not be efficient when a DSB occurs within a sequence that diverges more than ~2% in identity from any possible template for repair (15). Indeed, the three examples of HR identified in this study all displayed long stretches of complete sequence identity between the two chromosomal regions involved in the recombination events (Fig. 5). This finding has been confirmed by other studies that observed similar, albeit somewhat shorter, regions of sequence identity at sites of recombination within *var* genes (43–45). Given the extensive sequence diversity within *var*, *rifin*, *stevor*, and *Pfmc-2TM* genes, the chance that a randomly occurring DSB will occur precisely at a position with sufficient sequence identity to another position in the genome to serve as a template for HR is low. In the absence of efficient NHEJ, such DSBs would generally be lethal. However, telomere healing provides an alternative pathway for stabilizing DSBs that occur within subtelomeric domains, thus enabling parasites to survive DNA damage within these regions and maintain genome integrity. Chromosomes that have undergone telomere healing could later undergo HR when a subsequent DSB occurs within a sequence that shares identity with a region of a full-length subtelomeric domain. The resulting gene conversion event would reestablish typical chromosomal structure, including a full complement of clonally variant gene copies as well as TAREs (Fig. 6). When such events occur within the coding regions of the variant antigen-encoding genes, new genes are created, as demonstrated by the HR event that occurred within the subtelomeric region of chromosome 13 described here (Fig. 3B) and as has been observed in other studies (43–45). Of note, these recombination events retain reading frame and general gene structure. Evidence for telomere loss and potential healing events in field isolates indicates that this mechanism of chromosome stabilization occurs in naturally circulating parasites (46–48). This provides a model for mitotic diversification of these important gene families using both telomere healing to stabilize chromosome ends and HR when breaks occur within areas of sequence identity, thereby creating new chimeric genes and restoring complete subtelomeric regions. Telomere healing therefore provides a complementary method to HR for preserving the structure of chromosome ends. Together, these two pathways of DSB repair function to maintain genome integrity and chromosome stability in the absence of robust NHEJ and also drive the generation of diversity within the clonally variant multicopy gene families of *P. falciparum*.

## MATERIALS AND METHODS

### Parasite culture.

*P. falciparum* parasites were cultivated at a 5% hematocrit in RPMI 1640 medium (Corning Life Sciences, Tewksbury, MA), 0.5% AlbuMAX II (Invitrogen), 0.25% sodium bicarbonate, and 0.1 mg/ml gentamicin. Cultures were maintained at 37°C in an atmosphere of 5% oxygen, 5% carbon dioxide, and 90% nitrogen. Individual clonal lines were obtained by limiting dilution (49).

### Parasite irradiation.

Irradiation was administered using a Rad Source 2000 irradiator set at 160 kV/25 mA. To generate random DSBs, 3D7 parasites seeded at 0.5% were exposed to 100-Gy X-ray irradiation three times consecutively, being allowed to recover to normal growth between each irradiation exposure. The degree of subtelomeric damage was preliminarily assessed after each round of irradiation by assaying for *var* gene deletion by quantitative PCR using genomic DNA (gDNA) as the template and the *var*-specific PCR primer set described previously by Salanti and colleagues (50). The subclone chosen for whole-genome sequencing displayed loss of three subtelomeric *var* clusters in this assay.

### Genomic DNA isolation.

One hundred milliliters of cultured parasites at 5 to 8% parasitemia was harvested for isolation of gDNA. DNA was isolated and purified using phenol-chloroform extraction followed by ethanol precipitation, as previously described (51).

### SMRTbell library preparation.

Sequencing libraries were produced using the PacBio 20-kb library preparation protocol for high-molecular-weight gDNA obtained from clonal parasite lines, as previously described (28). We used the SMRTbell template prep kit 1.0 (Pacific Biosciences) following the standard 20-kb template preparation using the BluePippin size Selection system protocol (Pacific Biosciences). Briefly, parasite DNA was sheared twice for 1 min at 5,300 rpm in an Eppendorf 5424 centrifuge using a g-Tube (Covaris) followed by damage repair, end repair, and ligation of SMARTbell adapters. Unligated DNA was digested with exonucleases, and the libraries were size selected using a BluePippin pulsed-field gel electrophoresis instrument (Sage Science) to isolate fragments greater than 15 kb. Library concentration was measured with the Qubit fluorometer dsDNA BR assay kit (Life Technologies, Inc.), and fragment length distributions were generated using the 2200 TapeStation (Agilent). Sequencing primer and P6 polymerase were annealed to the libraries according to the manufacturer’s protocols (Pacific Biosciences) and performed with P6-C5 chemistry and v3 SMRT cells on an RSII instrument at Weill Cornell Medicine.

### Genome sequencing and analysis.

Pacific Biosciences RSII-based single-molecule sequencing was used to prepare long-read datasets suitable for accurate assembly of the subtelomeric regions of the parasite’s genome. Six RSII SMRTCells were used for the nonirradiated clone library, while the irradiated clone library was sequenced with eight SMRTCells. Filtered sequence data were assembled using HGAP 2.0 with Quiver polishing. This Celera Assembler-based *de novo* assembly approach produced genome sizes of 23.2 Mb and 23.3 Mb for the nonirradiated and irradiated clones, respectively. The largest contig was 3.2 Mb, and assembled contig N50 values were 1.5 Mb for both lines. The nonirradiated line generated 29 polished contigs, while the irradiated line produced 24 polished contigs. The average coverage for each assembly was 130× and 100× for the nonirradiated and irradiated lines, respectively, and the average consensus concordance for both assemblies was greater than 0.9975; therefore, only 2.5 bases in 1,000 would be assigned incorrectly.

In order to identify recombination break points, telomere additions, or telomere deletions, each assembly was first aligned to a 3D7 genomic sequence from PlasmoDB using Mauve (28). From the Mauve alignment, chromosomes were assigned to each polished contig in both assemblies. Thirty kilobases from the right and left ends of each chromosome from the irradiated and nonirradiated polished assemblies were then aligned to the PlasmoDB reference genome by using BLAST (52). Recombination break points and telomere additions were determined via manual inspection of the tabulated BLAST output. Each break point was also confirmed using the NCBI Blastn online tool (https://blast.ncbi.nlm.nih.gov/Blast.cgi).

To validate structural variants (SV) seen in comparisons between the PlasmoDB 3D7 reference and our assemblies, reads from the datasets used for assemblies of irradiated and nonirradiated clones were aligned to the Plasmo DB reference using BWA-MEM (version 0.7.13-r1126; arXiv:1303.3997) with default parameters. The resulting alignments were processed with SAMtools (version 2.6.32-279.el6.x86_64) (53) and visualized with IGV (version 2.3.92) for manual inspection of loci with predicted SVs. Analyses of the sequences upstream of the loci at which telomere repeats were added were performed using the MEME software suite and did not identify discernible motifs shared between any of the sequences.
