# Supplementary material for: Chromosome End Repair and Genome Stability in Plasmodium falciparum
Source: mBio. 2017 Aug 8;8(4):e00547-17. doi: 10.1128/mBio.00547-17 (PMC5550746; doi:10.1128/mBio.00547-17)
Supplement: FIG S1 [file mbo004173427sf1.pdf]

Telomere Healing Events on Chromosome 2 and 3

Sequence read coverage associated with Figure 2B

Chromosome 2L

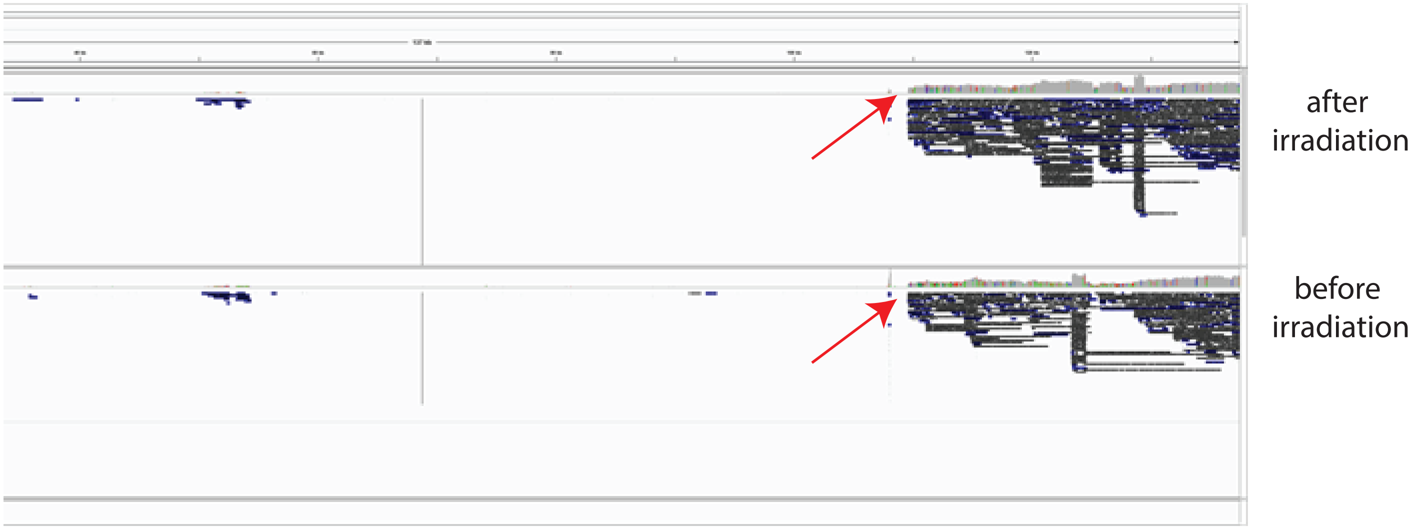

Chromosome 3

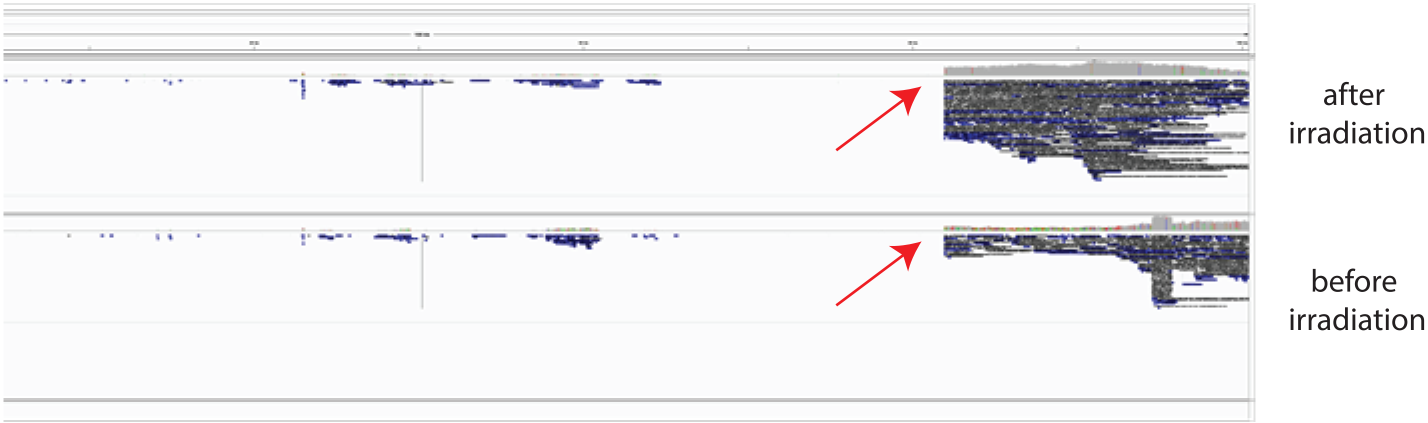

Supplemental Figure 1. Sequence read "pile-ups" displaying the detection of telomere healing events on chromosomes 2 and 3. These events are displayed schematically in Figure 2B of the main text. For both chromosomes, the reads obtained from the irradiated clone are shown on top and the reads obtained the non-irradiated clone are shown on the bottom. All reads are aligned with the 3D7 reference sequence obtained from Plasmodb.org. The sites of telomere healing are denoted with a red arrow. The subtelomeric deletions are detectable in both the irradiated and non-irradiated clones, indicating that these two healing events occurred during culture of the parent line prior to exposure to X-ray irradiation. Reads that align within the deleted regions in the irradiated line coincide with telomere associated repetitive elements (TAREs) that are shared between many subtelomeric domains.
