# Supplementary material for: Chromosome End Repair and Genome Stability in Plasmodium falciparum
Source: mBio. 2017 Aug 8;8(4):e00547-17. doi: 10.1128/mBio.00547-17 (PMC5550746; doi:10.1128/mBio.00547-17)
Supplement: FIG S3 [file mbo004173427sf3.pdf]

ATGAAGGGATCTGGATCAGAAAAAAATGTATATCTTTCAAATAAAAATAAAGAAATTAATATGAACCAACAATCAG
ATAATAAAATGTGTGATGAATGTGATGATATGAATCAACCAGGAGATGTAAATAAAAATGACAAAACATCAAATG
ATCAAGCAAATTCAAGTGATTCTGATTGTGAGCCCTTACCATTTGGATTAAAACCTTCAGATTTAAATAGAAAAGTT
ACAGAAGAAGATTTAGAAAGAATGATAATAGAATTACCAGGAAAATTAGAAAGGAAAGATATGTATTTAATATGG
CATTATAGTCATTCTCTTTTGAGAGATAAATTTAATAAAATGAAAAGTTCGTTATGGAGTATTTGTGGGAAATTAGC
TCATGAACATAAGTTACCATTCAAAATTAAAATGAAGAAATGGTGGAAATGTTGTGGTCATGTTACAGATGAATTA
TTAATAAAAGAGCATGATGATTATAATTCTATATATAATTATATTAATAATGAATCATCAAGTCGTGAACAATTTCTT
ATATTTCTTAATATGATAAAGCATTCATGGACAACATTTACTATGGAGACTTTTATTAAATGTAAGATTTCTTTAGAA
AATAACATGAGAAATGTTACAGGGTTTAGGGTTCAGGGTTTAGGGTTTAGGGTTCAGGGTTTAGGGTTTAGGGTT
TAGGGTTTAGGGTTTAGGGTTTAGGGTTTAGGGTTTAGGGTTCAGGGTTTAGGTTTAGGGTTTAGGGTTCAGGGT
TTAGGTTTAGGGTGTAGGGTTCAGGGTTTAGGGTTTAGGGTTCAGGGTTTAGGGTTTAGGGTTTAGGGTTTAGGG
TTTAGGGTTCAGGGTTTAGGGTTCAGGGTTTAGGGTTCCAGGGTTCAGGGTTCAGGGTTTAGGGTTCAGGGTTTA
GGGTTCAGGGTTTTAGGGTTCAGGGTTCAGGGTTTAGGGTTTAGGGTTCAGGGTTTAGGGTTCAGGGTTCAGGGT
TTAGGGTTTAGGGTTCAGGGTTTAGGGTTCAGGGTTCAGGGTTTAGGGTTCAGGGTTTAGGTTTTAGGTTTAGGG
TTCAGGGTTTAGGGTTTTAGGTTTAGGGTTCAGGGTTTAGGGTTCAGGGTTTAGGGTTTAGGGTTCAGGGTTCAG
GGTTTAGGGTTTAGGGTTCAGGGTTTAGGGTTCAGGGTTCAGGGTTTAGGGTTCAGGGTTTAGGGTTCAGGGTTC
AGGGTTTAGGGTTCAGGGTTTAGGGTTCAGGGTTCAGGTTTAGGGTTTAGGGTTTAGGGTTTAGG

Supplemental Figure 3. Assembled sequence showing the telomere healing event associated with the end of chromosome 2L, as shown schematically in Figure 2B of the main text. The coding region of the gene *lsap2* is shown in black text while the telomeric repeats are shown in blue.
