# Supplementary material for: Chromosome End Repair and Genome Stability in Plasmodium falciparum
Source: mBio. 2017 Aug 8;8(4):e00547-17. doi: 10.1128/mBio.00547-17 (PMC5550746; doi:10.1128/mBio.00547-17)
Supplement: FIG S4 [file mbo004173427sf4.pdf]

CCCTAAACCCTGAACCCTAAACCCTAAACCCTAAACCCTAAAACCCTGAACCCTAAACCTAAACCCTGAACCCTAAA
CCCTGAACCCTAAACCTAAACCCTAAACCCTAAACCCTGAACCTAAACCTAAACCCTGAACCCTAAACCCTAAACCC
TAAACCTAAACCCTAAACCCTAAACCCTGAAACCCTGAACCCTGAACCCTAAACCCTAAACCCTAAACCCTAAACCC
TGAACCCTAAACCTAAACCCTAAACCCTAAACCCTAAACCCTAAACCCTAAACCCTAAACCCTGAACCCTAAACCCT
GAAACCCTAAACCCTAAACCCTAAACCCTAAACCCTAAACCCTGAAACCCTAAACCTAAACCCTAAACCCTAAACCC
TGAACCCTGAACCCTAAACCCTGAACCCTAAACCCTGAACCCTAAACCCTGAAAAAAAAAAAAAAAAAAAAAAAGT
ATGTACAATGATAATAAATATATATATATATATAAAAAAAAAAAAAGGGTATAGACCACATAATATTATATTTTATTTTT
TTTTTTTCTTATATATTATGTGGTACATCCATTTTTATCAACAGGGAAATAAATATCATCTGATTTTAAATTCACATCA
TTTGGAAAAGTGGTAAAGATTGCAATACATAGAATAATATCTGAAGTTCTTGAAGGTATTATATAATGATTTTCTCC
TTTGAAAACAATAATATGAGAATTAGTAAAATAGGTATTTAAATTTTTATAAACATCGTCATCATAAAGATCATCTT
TTTCTCCACAAAATATTAAAGTAGGTATATTTTTTTTCCCTATTTCTATAAAAATGTCATGGGAACTCCACATTGGCA
TATTATTTAAACAACCATATATACAATCGGTTAAATTCTTTTTGGCAAAAGCATTCCACATTAATTTATCATAAACTA
CTTCAAAATCATTTCGTGGAATACAATGTTTCATACAACATTTGGGAAAACAAAATGGTGACATAATACATGAACA
AGAATTAATTATACTTAATAATCCATTTCTTATTTTTAATGATAAAGGTTTCCTTCCTAACATACCCACAGGTGATAA
AAAAACAATTTTTTTAACTTGATTAATATATTTCTGTGCAAATGCAGCAGCTATTAAACAACCCATTGAACCACCAAT
CAAATAAAATTCCTTATTTTGCAAATTTAAATAACATACTAATTCTTCTATTTGAGTCAAAAAAAAATTTAAATTATA
TACATCATCACTACTATATTTTGGACATTCAGATAACCCATGTCCATATAAATCATACTTAAGTATTTGATAATTATT

Supplemental Figure 4. Assembled sequence showing the telomere healing event associated with the end of chromosome 3, as shown schematically in Figure 2B of the main text. The coding region of *alpha/beta hydrolase* gene is shown in black text while the telomeric repeats are shown in blue. A short stretch of subtelomeric DNA between the coding region and the telomere repeats is shown in purple text.
