# Supplementary material for: Chromosome End Repair and Genome Stability in Plasmodium falciparum
Source: mBio. 2017 Aug 8;8(4):e00547-17. doi: 10.1128/mBio.00547-17 (PMC5550746; doi:10.1128/mBio.00547-17)
Supplement: FIG S5 [file mbo004173427sf5.pdf]

GAACCCTAAACCCTGAACCCTAAACCTAAAACACTAACCCTGAACCCCAAACCCTGAACCCTAAACCCTAAAACCTG
AACCCTAAACCGGAACCCTGAACCCTAAACCCTAAACCCTGAACCATAAACCCTGAACCCTAAACCTAAACCCTGA
ACCCTGAACCCTAAACCCTGAACCCTAAACCCTGAACCCTAAACCTAAACCCTAAACCTAAACCCTAAACCCTGAAA
CCCTAAACCCTGAACCCTAAACCCTGAACCCTAAACCCTAAACCCTGAACCCTAAACCCTGAAACCCTGAACCCTAA
ACCCTAAACCCTGAAACCCTGAAACCCTAAACCCTAAACCTAAACCCTGAACCCTAAACCCTGAACCCTAAACCCTG
AACCCTAAACCCTAAACCCTAAACCCTGAACCCTAAACCCTAAACCCTAAACCTAAACCCTGAACCCTAAACCCTAA
ACCCTAAAACCTGAACCCTGAACCCTGAAACCCTGAACCCTAAACCCTAAACCCTGAACACTACACCCTAAACCCTG
AACCCTAAACCCTAAACCCTGAAACCCTGAACCCTAAACCCTAAACCCTGAACCCTAAACCCTAAACCCTAAACCCT
AAACCCTGAACCCTGAACCCTAAACCCTGAAACCTTGAAACAGTACTCCGTTCAACGTTCACTGCAGCATCAGATTT
TACTTTCACCCAAGTGCAAAAAGTCTCACGAGCGCCAGAGAAATCAAAAAAACATGACGATGGGTCATATTTTTGA
ATTATATCACTAGAAATCTTTAACGCACTAGTATAATCCGTTGTACTAAAATAGGATACCAATCGCTGATCACCTAC
AGTAAATATACCCATTTTTTGTAATGCTGCAATAACAAATTCCTTACCTGCCTTAGTACCTGCGGCTTCACCCGCTTC
AACACTAGCCTCAATACCCTTTTGAATCGCAGTTACTAAAGCGGCGCTTCTCCAAGCATAGATACCCAATCCTCCGA
ATAATCCAATACTTGCTGCAACACCTCCTAGCCCACACCCACACCTAAGACAACCTTTTTCTACTTTTTCTGCAAATG
ATTTGTCCTTCCTATCTTTTTCAATAATTTCTTGTATATTTTTGTCACGTTCTTCTTTACGTTTTTGGCGTTTATCTTTAA
TACGTTCTTCGTATTCTTCAAAACGTTGCGACGCTTGTCGATCGAAAGTTCCCTTCACTGATTTCATATCCGCATCAT
TATCATATTTAGGCAT

Supplemental Figure 5. Assembled sequence showing the telomere healing event associated with the end of chromosome 1, as shown schematically in Figure 3A of the main text. The coding region of a *rif* gene is shown in black text while the telomeric repeats are shown in blue.
