# Supplementary material for: Chromosome End Repair and Genome Stability in Plasmodium falciparum
Source: mBio. 2017 Aug 8;8(4):e00547-17. doi: 10.1128/mBio.00547-17 (PMC5550746; doi:10.1128/mBio.00547-17)
Supplement: FIG S6 [file mbo004173427sf6.pdf]

ATGAAGGGATCTGGATCAGAAAAAAATGTATATCTTTCAAATAAAAATAAAGAAATTAATATGAACCAACAATCAG
ATAATAAAATGTGTGATGAATGTGATGATATGAATCAACCAGGAGATGTAAATAAAAATGACAAAACATCAAATG
ATCAAGCAAATTCAAGTGATTCTGATTGTGAGCCCTTACCATTTGGATTAAAACCTTCAGATTTAAATAGAAAAGTT
ACAGAAGAAGATTTAGAAAGAATGATAATAGAATTACCAGGAAAATTAGAAAGGAAAGATATGTATTTAATATGG
CATTATAGTCATTCTCTTTTGAGAGATAAATTTAATAAAATGAAAAGTTCGTTATGGAGTATTTGTGGGAAATTAGC
TCATGAACATAAGTTACCATTCAAAATTAAAATGAAGAAATGGTGGAAATGTTGTGGTCATGTTACAGATGAATTA
TTAATAAAAGAGCATGATGATTATAATTCTATATATAATTATATTAATAATGAATCATCAAGTCGTGAACAATTTCTT
ATATTTCTTAATATGATAAAGCATTCATGGACAACATTTACTATGGAGACTTTTATTAAATGTAAGATTTCTTTAGAA
AATAACATGAGAAATGTTACAGGGTTTAGGGTTCAGGGTTTAGGGTTTAGGGTTCAGGGTTTAGGGTTTAGGGTT
TAGGGTTTAGGGTTTAGGGTTTAGGGTTTAGGGTTCAGGGTTTAGGTTTAGGGTTTAGGGTTCAGGGT
TTAGGTTTAGGGTGTAGGGTTCAGGGTTTAGGGTTTAGGGTTCAGGGTTTAGGGTTTAGGGTTTAGGGTTTAGGG
TTTAGGGTTCAGGGTTTAGGGTTCAGGGTTTAGGGTTCAGGGTTTAGGGTTCAGGGTTTAGGGTTCAGGGTTTAG
GGTTCAGGGTTAGGGTTCAGGGTTTAGGGTTCAGGGTTTAGGGTTCAGGGTTTTAGGGTTCAGGGTTCAGGGTTT
AGGGTTTAGGGTTCAGGGTTTAGGGTTCAGGGTTCAGGGTTTAGGGTTCAGGGTTCAGGGTTTAGGGTTTAGGGT
TCAGGGTTTAGGGTTCAGGGTTCAGGGTTTAGGGTTCAGGGTTTAGGTTCAGGGTTTAGGTTTTAGGTTTAGGGT
TCAGGGTTTAGGGTTTTAGGTTTAGGGTTCAGGGTTTAGGGTTCAGGGTTTAGGGTTTAGGGTTCAGGGTTCAGG

Supplemental Figure 6. Assembled sequence showing the telomere healing event associated with the end of chromosome 2R, as shown schematically in Figure 3A of the main text. The coding region of *Pf3D7_0221000* is shown in black text while the telomeric repeats are shown in blue.
